# Biochemical Characterization of Ethiopian Black Cumin (*Nigella sativa* L.)

**DOI:** 10.1155/2024/2746560

**Published:** 2024-08-16

**Authors:** Basazinew Degu Gebremedin, Bizuayehu Tesfaye Asfaw, Wendawek Abebe Mengesha, Kebebew Assefa Abebe

**Affiliations:** ^1^ Wondo Genet Agricultural Research Center Ethiopian Institute of Agricultural Research, P. O. Box 198, Shashemene, Ethiopia; ^2^ School of Plant and Horticultural Science Hawassa University, P. O. Box 05, Hawassa, Ethiopia; ^3^ Department of Molecular Cellular and Microbial Biology Addis Ababa University, P. O. Box 3434, Addis Ababa, Ethiopia; ^4^ Debre Zeit Agricultural Research Center Ethiopian Institute of Agricultural Research, P. O. Box 32, Debre Zeit, Ethiopia

**Keywords:** association, cluster, essential oil, fixed oil, principal component

## Abstract

Black cumin (*Nigella sativa* L.) seed oil has been used for its medicinal and aromatic values. Some studies revealed the presence of variability among *N. sativa* genotypes in seed oil content and yield. In Ethiopia, very few studies were conducted to investigate the variability of *N. sativa* genotypes by using biochemical traits. Thus, this study was conducted at Debre Zeit and Kulumsa Agricultural Research Centers' experimental sites under field conditions during the 2021 cropping season to investigate the variability of Ethiopian *N. sativa* genotypes based on biochemical traits. Sixty-four genotypes were used and arranged in an 8 × 8 simple lattice design with two replications. Essential oils (EOs) and fixed oils were extracted by the respective methods of hydro distillation and solvent extraction. The univariate, bivariate, and multivariate analyses of the collected data were performed. Combined analysis of variance (ANOVA) revealed significant differences among genotypes in fixed oil yield per hectare (FOY), EO content (EOC), and EO yield per hectare (EOY). EOY had a significant positive correlation with FOY and EOC. It is expected to improve all biochemical traits by 17.39%–94.62% over the improved varieties by selection of the top 5% landraces. Therefore, genotypes 90504, 219970, and 013_ATH were the top 5% best performed landraces by FOY and EOY over the improved varieties. So, through selection, it would also be possible to improve the studied biochemical traits of the genotypes. The principal component (PC) analysis (PCA) of four biochemical traits showed 85.86% of the total variance captured by the first two PCs. EOY and FOY were the main contributor traits to the variation in the first PC, whereas FOC and EOC were the main contributor traits to the variation in the second PC. The genotypes were grouped into three different clusters based on four biochemical traits with significant intercluster distance. This showed that there was sufficient diversity among the genotypes which can be exploited for the future *N. sativa* improvement program in Ethiopia.

## 1. Introduction

Black cumin (*Nigella sativa* L.) is cultivated for its seed, fixed oils, and essential oils (EOs). Fixed oils are extracted from grounded black cumin seeds with an organic solvent which contains volatile and nonvolatile constituents. However, EOs are natural, secondary metabolites and volatile complex compounds characterized by the aroma of their corresponding aromatic plants [1–3].

The biochemical traits of *N. sativa* seeds vary depending on the extraction method, variety, maturity stage, and growing environment [4, 5]. As several studies showed, these products are used for the treatment of various ailments like Parkinson's [6–8], Alzheimer's [9–14], neuroinflammation [15–17], ischemic stroke [18, 19], traumatic brain injury [20], anxiety and depression [21, 22], epilepsy [23], and schizophrenia [24].

In Ethiopia, *N. sativa* has been cultivated and used for its spice and medicinal value. Currently, *N. sativa* seed fixed oil locally known as “Ye Tikur Azmud Zeyit” is well adapted and sold in many pharmaceuticals as a treatment of common cold. *N. sativa* is now a means of income for small-scale households by selling of whole seeds and seed fixed oil. The *N. sativa* oil market in the United States was valued at more than 15 million USD in 2018. It is anticipated that this amount will rise to about 25 million USD annually by 2025. Due to an increase in *N. sativa* plantations, the market for its oil in Asia Pacific, which is dominated by Australia, South Korea, Japan, China, and India, is expected to grow to a value of over 10 million USD by 2025 [25].

Evaluation and documentation of the existing genetic diversity are very important for maintenance and exploration of the variability of *N. sativa* breeding program. Different scholars reported the genetic diversity of *N. sativa* from many producing countries of the world [26–29]. In Ethiopia, the existence of genetic diversity of *N. sativa* seed oil was reported by researchers on a limited number of genotypes [30–33]. The highest mean FOC (43.24%) was reported by Fikre et al. [33], whereas the least mean value (18%) was reported by Kinki [30]. The highest (1.3%) and least (0.30%) mean EO content (EOC) was reported by Mengistu and Wegayehu [32] and Kinki [30], respectively.

Biochemical characterization is another alternative criterion for evaluation and documentation of genotypes. FOC, fixed oil yield per hectare (FOY), EOC, and EO yield per hectare (EOY) were analyzed separately for univariate and combined for bivariate and multivariate like cluster and principal components (PCs). Based on this result, there is a high level of genetic variability in *N. sativa* genotypes of Ethiopia that can be exploited for identification and selection of the spice and medicinal plant breeding program. This provides a baseline information for academia, researchers, industries, policy makers, and societies those rely on this crop.

As aforementioned, some authors reported the genetic diversity of few Ethiopian *N. sativa* collections by seed oil content and yield. This is the limitation that should be addressed by using many collections of *N. sativa* cultivated in the country to exploit the existing full potential. Therefore, this study is designed to evaluate the Ethiopian *N. sativa* genotypes by their seed oil content and yield.

## 2. Materials and Methods

### 2.1. Planting Materials and Experimental Procedures

**Figure 1:**
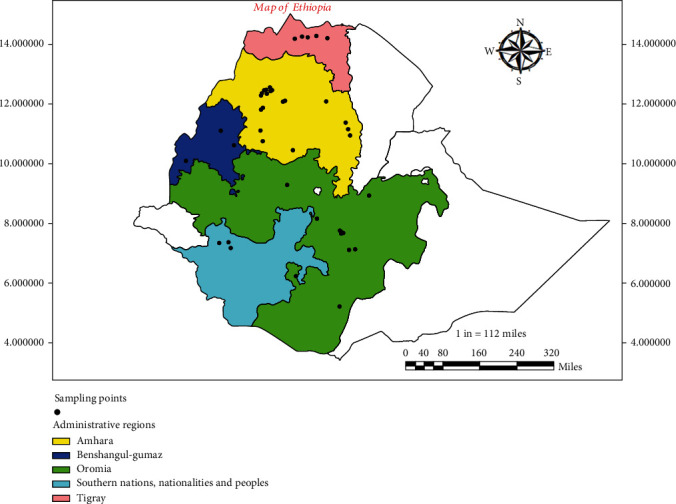
Map of Ethiopia showing collection sites of each *N. sativa* L. genotypes from different regions.

The improved varieties and accessions gathered from potential black cumin growing regions in Ethiopia, including Oromia, Amhara, Tigray, Benishangul-Gumuz, and Southern Nations and Nationality Peoples (SNNP) regions, were included in these genotypes (Figure 1). Unless they can be distinguished morphologically by appearance, the individual genotype that was initially obtained was collected from sites that were at least 5 km apart. The field experiment was carried out at Debre Zeit and Kulumsa Agricultural Research Centers in Ethiopia during the 2021 cropping season. An 8 × 8 simple lattice design with two replications was used to set up the experimental fields. Rows within the same plot, plots within the same block, plots within the same replication, and between replications were all separated by the corresponding distances of 30 cm, 1 m, 1 m, and 2 m. The plots were 2 m by 1.5 m in length and width. The seeds of each genotype were sown directly onto the field at a soil depth of 3 cm after the field was well prepared at a seed rate of 15 kg ha^−1^. Sowing was made at the beginning of main rainy season. When necessary, appropriate crop management practices including weeding, hoeing, and thinning were used. ArcGIS Desktop Advanced Version 10.8 software was used to create the map [34].

### 2.2. Data Collection

Biochemical data such as FOC (percent), FOY (kilogram), EOC (percent), and EOY (kilogram) were critically recorded from the collected dried seeds of *N. sativa* by using the following procedures.

#### 2.2.1. Fixed Oil (Crude Fat) Content (Percent)

The FOC of the samples was determined using the Soxtec Extraction System (Foss Soxtec™ 8000 Extraction Unit, Sweden) according to the AOAC [35] method. Three grams of composite dried and crushed *N. sativa* seed samples from each genotype was weighed into thimbles lined with cotton at the bottom. The mass of the cooled cups was then measured. The thimbles containing the samples were placed into the Soxtec™ 8000 Extraction System, and 50 mL of n-hexane was added as a solvent to each cup using a dispenser. The extraction process consisted of 20 min of boiling, 30 min of rinsing, and 10 min of recovery. The cups with their residue were then removed from the Soxtec System and placed in a drying oven at 105°C for 30 min. The cups were subsequently cooled in desiccators for an hour. The mass of each cooled cup together with its FOCs was weighed. The FOC was calculated by the following formula [36]:

(1) $$\begin{array}{c} \text{FOC}\left(\frac{w}{w},\%\right)=\frac{\text{weight of fixed oil }\left(\text{g}\right)}{\text{weight of sample }\left(\text{g}\right)}\times 100 \end{array}$$

#### 2.2.2. FOY (Kilogram)

FOY is the fixed oil yield obtained from the ground *N. sativa* seeds that was harvested from the central rows of each plots and converted into yield per hectare based on FOC and seed yield per hectare. It was determined by the following formula:

(2) $$\begin{array}{c} \text{FOY }\left(\text{kg}\right)=\frac{\text{FOC }\left(w/w,\%\right)\times \text{seed yield per hectare }\left(\text{kg}\right)}{100} \end{array}$$

#### 2.2.3. EOC (Percent)

**Figure 2:**
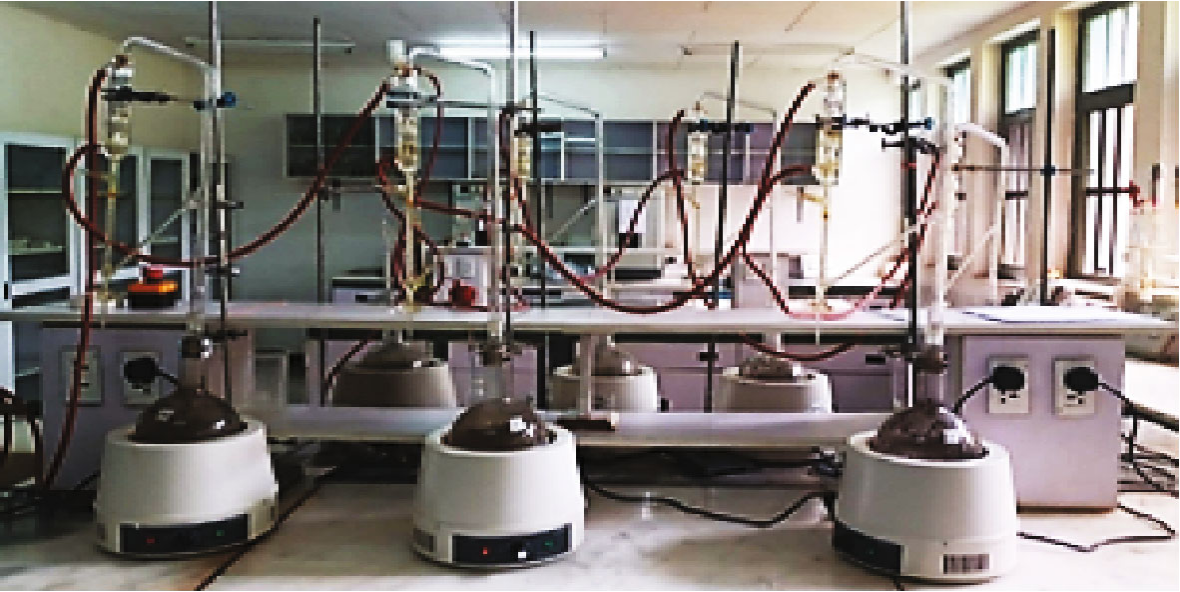
The assembled Clevenger apparatus setups.

**Figure 3:**
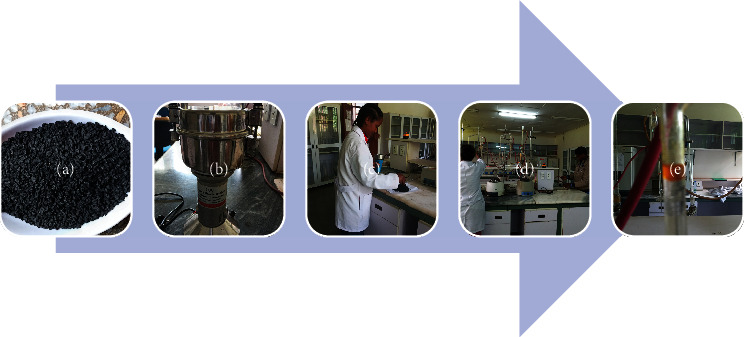
Essential and fixed oil extraction process: (a) *N. sativa* seeds; (b) seed grinder; (c, d) grinded the seed, put it in the flask containing water, and assembled the Clevenger apparatus, respectively; (e) the extracted essential oil in the flask.

In order to determine EOC (*w*/*w*, %), 100 g of composite ground *N. sativa* seed samples was taken and put in 0.5 L water from each genotype separately and then subjected to hydrodistillation for 3 h using Clevenger apparatus (Figure 2) at Wondo Genet Agricultural Research Center, Natural Products Laboratory (Figure 3), according to the standard procedure described by Baj et al. [37]. The amount of extracted EO was measured by using measuring pipette. EOC was determined by the following formula [36]:

(3) $$\begin{array}{c} \text{EOC }\left(\frac{w}{w},\%\right)=\frac{\text{weight of oil }\left(\text{g}\right)}{\text{weight of sample }\left(\text{g}\right)}\times 100 \end{array}$$

#### 2.2.4. EOY (Kilogram)

EOY is the EO yield obtained from the ground *N. sativa* seeds that was harvested from the central rows of each plots and converted into yield per hectare based on EOC and seed yield per hectare. It was determined by the following formula:

(4) $$\begin{array}{c} \text{EOY }\left(\text{kg}\right)=\frac{\text{EOC }\left(\text{w}/\text{w},\%\right)\times \text{seed yield per hectare }\left(\text{kg}\right)}{100} \end{array}$$

### 2.3. Data Analysis

Prior to conducting the analysis, the validity of each individual experiment was evaluated by using Bartlett's test to determine the homogeneity of error variances for each of the measured traits. As a result, it was discovered that most of the parameters were similar between the two sites. Next, the combined analysis of variance (ANOVA) of FOC, FOY, EOC, and EOY data from the two locations was carried out based on the model of simple lattice design proposed by A. Gomez and K. Gomez [38] using SAS Version 9.4 computer software programs [39]. The PROC GLM procedure was utilized to conduct an ANOVA in order to address the imbalance of treatments in the combined analysis. To find significant differences between genotype means, the least significant difference (LSD) test procedure was employed at a 5% significance level. Genotypes were regarded as fixed variables in the analysis, whereas replications, blocks, and locations were regarded as random variables.

PC analysis (PCA) and cluster analysis were applied to the mean of the genotype data that were collected using R software Version 4.2.2 [40]. The R software packages “factoextra” [41], “cluster” [42], “class” [43], and “clv” [44] were used to carry out the hierarchical cluster analysis. The aggregation patterns of the 64 *N. sativa* genotypes and populations that were selected based on their similarity to the corresponding means of all the collected traits examined by using clustering. Using the R software package “Nbclust,” the ideal number of clusters for the data set was ascertained [49]. Euclidean distance was used to calculate the genetic distance, and the ward D2 linkage method was used to create the dendrogram using the distance matrix. Intercluster distance was calculated based on the standardized Mahalanobis's *D*^2^ statistics [45] as

(5) $$\begin{array}{c} {D^{2}}_{ij}={\left(xi-xj\right)}^{\prime }{\mathrm{cov}}^{-1}\;\left(xi-xj\right) \end{array}$$

where *D*^2^_*ij*_ is the distance between cases *i* and *j*, *xi* and *xj* are vectors of the values of the variables for cases *i* and *j*, and cov^−1^ is the pooled within group variance-covariance matrix. The significance of *D*^2^ values between any two clusters was tested both at 1% and 5% probability levels against the tabulated chi-square (*χ*^2^) values at *p* − 1 degrees of freedom where *p* refers to the number of quantitative characters considered [46].

The genotypes with higher levels of desirable traits were selected for further production and improvement programs, and clusters containing these genotypes were identified. The R software packages “factoextra,” “ggplot2” [47], “corrplot” [48], and “ggsignif” [49] were utilized in conjunction with PCA to determine the traits that account for a significant portion of the overall variation between the populations and groups.

## 3. Results and Discussion

### 3.1. Univariate and Descriptive Analysis

Combined ANOVA over the two locations revealed a significant (*p* ≤ 0.001) effect for location and genotypes in FOY, EOC, and EOY. Supporting result was reported by Fikre et al. [33] on black cumin FOC. However, FOC was nonsignificant (*p* > 0.05) in the case of location. Location × genotype interaction effects were also significant (*p* ≤ 0.001) in FOC, FOY, EOC, and EOY (Table 1). Supporting result was reported by Mengesha and Alemaw [50] on coriander EOC and FOC.

Separate analyses of variance showed significant differences among the genotypes by FOC, FOY, EOC, and EOY. Similarly, combined analyses of variance exhibited significant differences in those measured biochemical traits. Significant variability was also detected from the wider ranges observed between the minimum and the maximum values of the traits measured (Table 2).

The variability of the genotypes for EOY and FOY creates a great opportunity for developing high-yielding varieties by improving the traits that are associated with them. This result would help the breeders to develop improved and suitable varieties for different agroecology of the country.

### 3.2. Mean Performance of the Genotypes on Biochemical Traits

Based on the combined result, there was a significant (*p* ≤ 0.001) difference among the evaluated genotypes for all biochemical traits studied (Table 2).

The FOC ranged from 30.1% to 46.47%, with a mean value of 37.89% (Table 2). The highest mean FOC was obtained from Genotype 19884 (from SNNP) followed by Genotype 9068 (from Amhara) and the improved variety DERSHYE, whereas the least mean value was obtained from Genotype 242840 (from Oromia) (Table 3). Studies from different countries reported *N. sativa* FOC was below this range [26, 30, 51–54] and within this range [27–29, 33, 55–58].

FOY ranged from 76.04 to 788.18 kg, with a mean value of 334.51 kg (Table 2). The highest mean FOY was obtained from Genotype 90504 (from Oromia) followed by Genotypes 013_ATH (from Amhara) and 219970 (from Tigray), whereas the least value was obtained from Genotype 237989 (from Oromia) (Table 3). Abdou et al. [59], Bayati, Karimmojeni, and Razmjoo [26], Hamed, Toaima, and Abd El-Aleem [53], Hosseini et al. [29], Rezaei-Chiyaneh et al. [54] and SALAHELDIN et al. [60] reported that *N. sativa* seeds FOY was found within this range. Seed yield per hectare is the main determinant of FOY.

EOC ranged from 0.09% to 0.83%, with a mean value of 0.35% (Table 2). The highest mean EOC was obtained equally from Genotypes 90504 (from Oromia) and 90501 (from Amhara) followed by Genotypes 215319 (from Amhara) and 229808 (from Benishangul-Gumuz), whereas the least mean value was obtained equally from Genotype 240404 (from SNNP) and the improved variety Silingo (Table 3). This might be resulted from the variations in the genetic make-up of the genotypes. Results from different countries reported that *N. sativa* EOC was within this range [26, 30, 54–56, 61].

EOY ranged from 0.41 to 9.75 kg, with a mean value of 3.2 kg (Table 2). The highest mean EOY was obtained from Genotype 90504 (from Oromia) followed by Genotypes 219970 (from Tigray) and 013_ATH (from Amhara), whereas the least mean value was obtained from Genotype 240404 (from SNNP) (Table 3). Abdou et al. [59] from Egypt and Bayati, Karimmojeni, and Razmjoo [26] and Hosseini et al. [29] from Iran reported that *N. sativa* EOY was within this range. Like FOY, seed yield per hectare is the main determinant of EOY. They have direct relationship.

The presence of variability among the *N. sativa* genotypes was observed by the wide range of biochemical traits studied. This indicates that there is a possibility of improving these traits through selection. Selection of the top 5% genotypes is predicted to improve biochemical traits by 17.39%–94.62% through selection (Table 4). Genotypes 90504 (from Oromia), 219970 (from Tigray), and 013_ATH (from Amhara) were the top 5% best performed landraces over improved varieties selected for FOY and EOY (Table 3). Gebremedin et al. [62] reported as Genotypes 90504 and 219970 were the top best performed landraces over the improved varieties in seed yield per hectare.

### 3.3. Relationship Among Biochemical Traits

Information on the nature and extent of association between any two characters is provided by correlation studies [63]. To measure the degree and direction of the relationship between biochemical traits, correlation analysis was made (Table 5). EOY had a significant positive correlation with FOY (*r* = 0.83^∗∗∗^) and EOC (*r* = 0.69^∗∗∗^); the association between EOC and EOY with FOC was nonsignificant. Similar relationships between FOY and EOC were reported by Hosseini et al. [29] on black cumin.

### 3.4. Cluster Analysis

According to Charrad et al. [64], the best number of clusters for the data set was determined, which was three.

**Figure 4:**
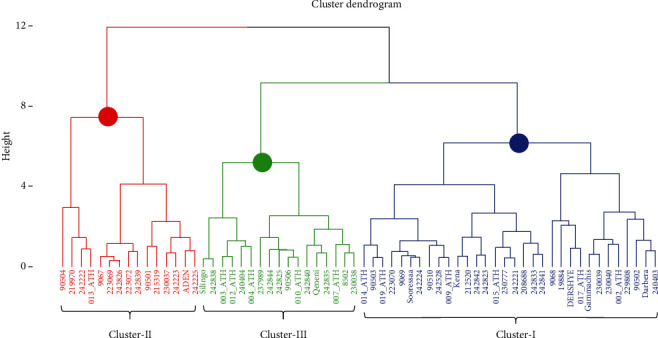
Dendrogram showing the relationships among the 64 *N. sativa* genotypes of Ethiopia evaluated for four biochemical traits.

Cluster I: This cluster was the largest group having 32 (50%) genotypes and consists of accessions from all collection regions and most of the improved varieties (Figure 4 and Table 6). This group was characterized by the highest FOC with the mean value of 39.09% (Table 7). Three of the 32 genotypes recorded above 43% of FOC: 19884 (44.97%), 9068 (44.21%), and DERSHYE (43.23%) (Table 3).

Cluster II: This group contained 15 (23.44%) genotypes and consists of accessions from Oromia, Amhara, Tigray, and Benishangul-Gumuz regions and only one of the improved varieties (Figure 4 and Table 6). This group was characterized by the highest FOY, EOC, and EOY with the mean value of 367.86 kg, 0.45%, and 4.69 kg, respectively (Table 7). Three of the 15 genotypes recorded above 495 kg of EOY: 90504 (582.03 kg ha^−1^), 013_ATH (509.28 kg ha^−1^), and 219970 (499.95 kg ha^−1^) (Table 3).

Cluster III: The remaining 17 genotypes (26.56%) from the five regions (SNNP, Oromia, Amhara, B/Gumuz, and Tigray) and two of the improved varieties (Silingo and Qeneni) belonged to this group and are characterized by the least mean values of all the studied biochemical traits (Figure 4 and Table 6).

The genotypes from Amhara, Oromia, and Tigray regions and the improved varieties were spread into all the three clusters but in different proportions (Table 8). However, the genotypes from SNNP and Benishangul-Gumuz regions were spread only into two clusters with different proportions. Most of the genotypes from Amhara, Oromia, SNNP, Benishangul-Gumuz regions and improved varieties were grouped under the first cluster (Table 8).

### 3.5. Intercluster Distance Analysis

Significant (*p* ≤ 0.001) intercluster genetic distances were observed between Clusters I and II (*D*^2^ = 634.78), Clusters I and III (*D*^2^ = 555.05), and Clusters II and III (*D*^2^ = 2054.45) which were also significant (*p* ≤ 0.001). The result shows the presence of high genetic divergence among genotypes within and between the clusters (Table 9).

**Figure 5:**
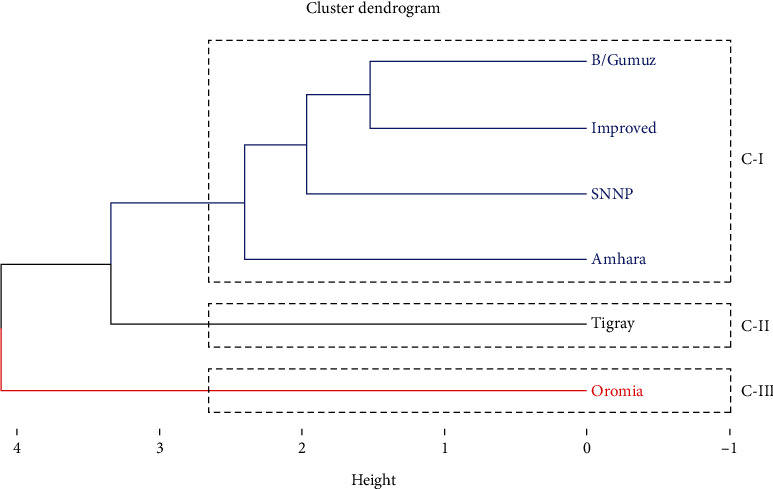
Dendrogram showing the relationships among the six groups of black cumin genotypes evaluated for four biochemical traits (blue, black, and red colors represent Clusters I, II, and III, respectively).

A dendrogram of the regions of origin was created using cluster analysis, which was based on means for groups of origin for four biochemical traits (Figure 5). Three groups of genotypes were identified. Benishangul-Gumuz, SNNP, Amhara, and improved cultivar genotypes made up the first cluster; genotypes from Tigray and Oromia were grouped into the second and third clusters, respectively. The close relationships between the genotypes in each cluster were amply displayed by the dendrogram. The highest mean value of FOY (343.74 kg) and the intermediate mean values of FOC (38.69%), EOC (0.33%), and EOY (3.12 kg) were the characteristics of the first cluster (Table 10). This suggests that this group's genotypes have a higher FOY than the others. Out of all the biochemical traits measured, the second cluster had the lowest mean value. In contrast, the third group had the highest FOC (39.03%), EOY (3.65 kg), EOC (0.40%), and intermediate in FOY (with a mean value of 339.38 kg) (Table 10).

### 3.6. PCA

The PCA grouped the four biochemical traits into four PCs, which explained the entire 100% of the variability among the studied genotypes. The first two PCs explained 85.86% of the variation that existed among the studied genotypes (Table 11).

An eigenvalue greater than one indicates that PCs account for more variance than accounted by one of the original variables in standardized data commonly used as a cutoff point for which PCs are retained [65]. The number of components is determined at the point, beyond which the remaining eigenvalues are relatively small and of comparable size [66, 67]. Therefore, based on the eigenvalues, two PCs having eigenvalues between 1.17 and 2.26 extracted a cumulative variance of about 85.86% of the total phenotypic diversity maintained (Table 11).

The influential PCs for clustering were the characters with the largest absolute values closer to unity than those with lower absolute values closer to zero [68]. So, due to the main contribution of the variations in FOY and EOY, the first PC explained up to 56.61% of the total variance (Table 11). However, the second PC explained about 29.24% of the total variance by the main contribution of FOC and EOC. The PCA confirmed that the collected Ethiopian *N. sativa* genotypes have high diversity, and most of the traits considered seemed to have high contributions toward the total phenotypic variability.

## 4. Conclusions

The result revealed the existence of significant variation for the studied biochemical traits among Ethiopian *N. sativa* genotypes. The mean performance of the genotypes discovered the wider ranges between the least and greatest values of all biochemical traits; this showed the presence of significant variation among *N. sativa* genotypes included in this study. This might be resulted from the difference in the genetic make-up of the genotypes. EOY had a significant positive correlation with FOY and EOC, but it did not correlate with FOC. FOY had a strong positive and significant correlation with FOC. About 85.86% of the variation that occurred among the studied genotypes was explained by the first two PCs. EOY and FOY were the main contributor traits for most of the variation occurring among the genotypes in the first PC, whereas FOC and EOC were the main contributor traits for the second PC. The PCA inveterates the existence of high variability among *N. sativa* genotypes of Ethiopia in all the biochemical traits studied. Genotypes of *N. sativa* were partitioned into three distinct clusters with significant variation in the distance among genotypes within and between them. Genotypes 90504, 219970, and 013_ATH were the top 5% best performed landraces over improved varieties selected for FOY and EOY. In general, this study confirmed the presence of enough variation in most of the biochemical traits studied in Ethiopian *N. sativa* genotypes which can create an enabling environment for the breeders to design active genotype collection, conservation, and use strategies. Moreover, this finding has a significant advantage for academia, industries, researchers, policy makers, and societies at large. Finally, it is recommended to conduct further evaluation studies on wider agroecological conditions of the country to enhance the improvement strategies by exploiting the existing diversity.

## Figures and Tables

**Table 1:** Combined analysis of variance of four biochemical traits recorded on 64 *N. sativa* genotypes of Ethiopia at Debre Zeit and Kulumsa during 2021 cropping season.

**Traits**	**Mean square**			**CV (%)**
	**Location (** **D** **F** = 1**)**	**Genotype (** **D** **F** = 63**)**	**Location** ^∗^ **genotype (** **D** **F** = 63**)**	
FOC (%)	1.80 ns	31.90^∗∗∗^	3.80^∗∗∗^	0.81
FOY (kg)	3400241^∗∗∗^	19813.23^∗∗∗^	11402^∗∗∗^	17.68
EOC (%)	0.38^∗∗∗^	0.03^∗∗∗^	0.03^∗∗∗^	11.04
EOY (kg)	512.12^∗∗∗^	4.89^∗∗∗^	2.75^∗∗∗^	18.75

Abbreviations: CV = coefficient of variation, DF = degree of freedom, EOC = essential oil content, EOY = essential oil yield per hectare, FOC = fixed oil content, FOY = fixed oil yield per hectare, ns = nonsignificant at *p* ≤ 0.05.

^***^Significant at *p* ≤ 0.001.

**Table 2:** Descriptive statistics, *F*-test, and coefficient of variation of four biochemical traits of the 64 black cumin genotypes of Ethiopia at each testing site and pooled during 2021 cropping season.

**Location**	**Statistics**	**FOC (%)**	**FOY (kg)**	**EOC (%)**	**EOY (kg)**
Debre Zeit	Mean ± SE	37.89 ± 0.31	449.76 ± 11.52	0.39 ± 0.01	4.61 ± 0.18
	Range	30.10–46.47	108.55–788.18	0.09–0.65	0.64–9.75
	*F*-test	^∗∗∗^	^∗∗∗^	^∗∗∗^	^∗∗∗^
	CV (%)	0.89	17.22	11.53	16.77

Kulumsa	Mean ± SE	37.72 ± 0.24	219.27 ± 6.21	0.31 ± 0.01	1.78 ± 0.08
	Range	32.88–43.69	76.04–410.23	0.09–0.83	0.41–5.88
	*F*-test	^∗∗∗^	^∗∗∗^	^∗∗∗^	^∗∗∗^
	CV (%)	0.68	11.56	8.81	11.31

Combined	Mean ± SE	37.81 ± 0.20	334.51 ± 9.73	0.35 ± 0.01	3.20 ± 0.13
	Range	30.10–46.47	76.04–788.18	0.09–0.83	0.41–9.75
	*F*-test	^∗∗∗^	^∗∗∗^	^∗∗∗^	^∗∗∗^
	CV (%)	0.81	17.68	11.04	18.75

Abbreviations: EOC = essential oil content, EOY = essential oil yield per hectare, FOC = fixed oil content, FOY = fixed oil yield per hectare, ns = nonsignificant at *p* ≤ 0.05, SE = standard error.

^***^Significant at *p* ≤ 0.001.

**Table 3:** Mean performance of four biochemical traits in 64 *N. sativa* genotypes of Ethiopia tested at Debre Zeit and Kulumsa during 2021 cropping season.

**Genotype**	**Biochemical traits**			
	**FOC (%)**	**FOY (kg)**	**EOC (%)**	**EOY (kg)**
Darbera	40.46	345.60	0.40	3.80
DERSHYE	43.23	349.87	0.33	2.68
ADEN	36.22	417.83	0.43	5.15
Sooressaa	39.10	362.79	0.31	3.20
Gammachis	38.82	292.12	0.38	3.02
Silingo	34.63	258.51	0.18	1.61
Kena	33.81	335.32	0.30	3.12
Qeneni	33.74	270.14	0.35	2.71
8502	34.25	211.37	0.40	2.65
9067	33.70	319.77	0.38	3.49
9068	44.21	298.56	0.23	1.49
9069	38.16	344.37	0.33	3.51
19884	44.97	456.67	0.25	2.70
90501	35.45	346.35	0.55	5.16
90502	42.63	345.36	0.47	3.68
90503	39.07	457.43	0.30	4.05
90504	42.89	582.03	0.55	7.18
90506	35.51	247.00	0.33	2.27
90510	37.86	381.49	0.35	4.35
208688	35.16	419.16	0.24	2.86
212520	34.97	324.56	0.29	2.83
215319	38.14	336.49	0.54	4.69
219970	40.18	499.95	0.49	5.91
223069	33.52	311.42	0.41	4.01
223070	38.51	347.25	0.38	3.44
223072	34.28	289.70	0.45	3.91
229808	40.18	263.10	0.53	2.17
230037	38.45	397.28	0.43	4.77
230038	35.64	248.34	0.40	2.74
230039	39.08	275.88	0.35	2.50
230040	41.11	233.07	0.40	2.63
230777	37.81	344.49	0.25	2.38
237989	35.13	185.97	0.35	1.81
240403	40.39	291.72	0.40	3.38
240404	39.98	272.60	0.18	1.13
242221	38.11	351.85	0.27	2.76
242222	40.37	455.81	0.39	5.18
242223	39.97	371.52	0.45	4.63
242224	39.32	363.72	0.35	3.37
242225	36.31	367.86	0.45	4.69
242528	39.57	388.68	0.38	3.75
242825	37.47	257.31	0.28	1.95
242826	33.98	324.05	0.40	3.81
242833	35.97	376.01	0.30	3.11
242835	34.47	263.35	0.30	2.50
242838	34.49	241.54	0.20	1.41
242839	32.96	315.43	0.45	4.48
242840	32.55	215.21	0.30	2.29
242841	37.39	384.81	0.30	2.71
242842	35.99	340.29	0.35	3.04
242844	35.70	274.40	0.30	1.42
242823	36.59	309.52	0.38	3.22
002_ATH	40.86	243.50	0.31	2.13
003_ATH	37.90	276.19	0.23	1.47
004_ATH	40.16	299.27	0.25	1.74
007_ATH	33.12	252.02	0.40	3.50
009_ATH	40.13	404.61	0.35	3.64
010_ATH	36.29	257.06	0.30	2.13
012_ATH	37.13	258.44	0.20	1.38
013_ATH	41.85	509.28	0.40	5.35
014_ATH	40.88	448.80	0.28	3.12
015_ATH	39.22	384.54	0.22	2.24
017_ATH	40.93	377.05	0.33	3.02
019_ATH	38.94	433.25	0.31	3.72
Mean	37.81	334.51	0.35	3.20
LSD (5%)	2.75	150.88	0.24	2.34
CV (%)	0.81	17.68	11.04	18.75

Abbreviations: CV = coefficient of variation, EOC = essential oil content, EOY = essential oil yield per hectare, FOC = fixed oil content, FOY = fixed oil yield per hectare, LSD = least significant difference.

**Table 4:** Comparison of mean performances of 5% of the best performed landraces selected for four biochemical traits over mean performance of improved varieties.

**Traits**	**Mean values**		**A comparative advantage over mean values of improved varieties (%)**
	**Top 5% landraces**	**Improved varieties**	
FOC (%)	44.02	37.5	17.39
FOY (kg)	530.42	329.02	61.21
EOC (%)	0.54	0.33	63.64
EOY (kg)	6.15	3.16	94.62

**Table 5:** Correlation coefficient of the four biochemical traits of 64 black cumin genotypes.

**Variables**	**FOC (%)**	**FOY (kg)**	**EOC (%)**	**EOY (kg)**
FOC (%)	1			
FOY (kg)	0.25^∗∗∗^	1		
EOC (%)	0.07 ns	0.26^∗∗∗^	1	
EOY (kg)	0.10 ns	0.83^∗∗∗^	0.69^∗∗∗^	1

Abbreviation: ns = nonsignificant at *p* ≤ 0.05.

^***^Significant at *p* ≤ 0.001.

**Table 6:** Clustering of 64 *N. sativa* genotypes of Ethiopia into three clusters using mean of four biochemical traits.

**Cluster**	**Number of genotypes**	**Genotypes included**	**Collection region**
I	32 (50%)	Kena, Gammachis, 230039, 208688, 90503, 242841, 230040, 242823, 242833, 242842, 009_ATH, 019_ATH, DERSHYE, 240403, Darbera, 242224, 19884, Sooressaa, 017_ATH, 014_ATH, 230777, 015_ATH, 242528, 9068, 223070, 229808, 212520, 90510, 9069, 002_ATH, 90502, 242221	Improved, SNNP, Oromia, Amhara, B/Gumuz, Tigray
II	15 (23.44%)	90504, 219970, 242222, 013_ATH, 9067, 223069, 242826, 223072, 242839, 90501, 215319, 230037, 242223, ADEN, 242225	Improved, Oromia, Amhara, Tigray, B/Gumuz
III	17 (26.56%)	Silingo, 242838, 003_ATH, 012_ATH, 240404, 004_ATH, 237989, 242844, 242825, 90506, 010_ATH, 242840, Qeneni, 242835, 007_ATH, 8502, 230038	Improved, Amhara, SNNP, Oromia, Tigray

**Table 7:** Mean value of four biochemical traits of 64 *N. sativa* genotypes of Ethiopia in each cluster.

**Trait**	**Cluster**		
	**I**	**II**	**III**
FOC (%)	39.09	36.31	35.51
FOY (kg)	348.56	367.86	257.31
EOC (%)	0.33	0.45	0.30
EOY (kg)	3.08	4.69	1.95

**Table 8:** Clustering of 64 *N. sativa* genotypes based on collection groups.

**Groups**	**Cluster**			**Total**
	**I**	**II**	**III**	
Amhara	11	7	6	24
Oromia	8	3	7	18
Tigray	2	3	1	6
SNNP	3	—	1	4
B/Gumuz	3	1	—	4
Improved	5	1	2	8
Total	32	15	17	64

**Table 9:** Average intercluster distance among clusters in *N. sativa*.

**Cluster**	**I**	**II**
I		
II	634.78^∗∗∗^	
III	555.05^∗∗∗^	2054.45^∗∗∗^

*Note:χ* ^2^ = 7.82, 11.35, and 16.27 at 5%, 1%, and 0.1% probability levels, respectively.

^***^Highly significant at *p* ≤ 0.001.

**Table 10:** The mean value of four biochemical traits of the groups of black cumin genotypes was evaluated during the 2021 cropping season.

**Trait**	**Cluster**		
	**I**	**II**	**III**
FOC (%)	38.69	35.02	39.03
FOY (kg)	343.74	293.07	339.38
EOC (%)	0.33	0.32	0.40
EOY (kg)	3.12	2.63	3.65

**Table 11:** Eigenvalues and eigenvectors of the first two principal components (PCs) for four biochemical traits of 64 *N. sativa* genotypes of Ethiopia.

**Variable**	**Principal components**	
	**PC1**	**PC2**
Eigenvalue	2.26	1.17
Proportion of variance (%)	56.61	29.24
Cumulative variance (%)	56.61	85.86
	Eigenvectors	
FOC (%)	−0.28	0.74
FOY (kg)	−0.55	0.35
EOC (%)	−0.48	−0.51
EOY (kg)	−0.63	−0.25

## Data Availability

The data are already included in the manuscript.
